# Voice Familiarization Training Improves Speech Intelligibility and Reduces Listening Effort

**DOI:** 10.1177/23312165251401318

**Published:** 2025-12-08

**Authors:** Freja Baxter, Harriet J. Smith, Emma Holmes

**Affiliations:** 1 Division of Psychology and Language Sciences, Department of Speech Hearing and Phonetic Sciences, 145281 University College London, London, UK

**Keywords:** voice familiarity, speaker familiarity, speech perception, cocktail party listening, effort, pupillometry

## Abstract

Understanding speech among competing speech poses a substantial challenge. In these environments, familiar voices—including naturally familiar (e.g., friends, partners) and lab-trained voices—are more intelligible than unfamiliar voices. Yet, whether familiar voices also require less effort to understand is currently unknown. We trained 20 participants to become familiar with three voices, then tested listening effort during a speech intelligibility task. During familiarization and training, participants were exposed to three talkers for different lengths of time, either speaking 88, 166, or 478 sentences (“Least Familiar,” “Moderately Familiar,” or “Most Familiar” voice, respectively). During each trial of the speech intelligibility task, two competing sentences were presented at a target-to-masker ratio (TMR) of −6 or +3 dB. Participants reported target sentences that were spoken by trained or by novel, unfamiliar talkers. We assessed effort using self-reported ratings and physiologically, using pupil dilation. We found that self-report scores were more sensitive than pupil dilation to differences in TMR, with lower self-reported effort at +3 than −6 dB TMR. The two measures may also be differentially sensitive to the extent of training. We found lower self-reported effort for all three trained voices over unfamiliar voices, with no differences among the trained voices, whereas pupil dilation was only lower for the voice that had been trained for the longest. Thus, both self-report scores and pupil dilation showed advantages for the voice that was trained for the longest (∼1 h), but self-report scores additionally showed reduced effort even following relatively short durations of training (<10 min).

## Introduction

In many everyday situations, understanding speech is fundamental for successful communication. Speech perception can occur effortlessly in quiet settings, but can be very effortful in adverse listening conditions, such as when competing talkers are present (Koelewijn et al., 2012). Known as the “cocktail party problem” (Cherry, 1953), the challenge of comprehending speech among competing speech is particularly demanding for older adults and for people with hearing loss (Duquesnoy, 1983; Marrone et al., 2008)—and substantial effort can be required to maintain accurate speech intelligibility in these settings (Pichora-Fuller et al., 2016). Previous studies have demonstrated that being familiar with a talker's voice can substantially improve speech intelligibility when competing speech is present (Barker & Newman, 2004; Buntrock et al., 2021; Case et al., 2018; Domingo et al., 2019, 2020; Holmes et al., 2021; Holmes & Johnsrude, 2020, 2021, 2023; Holmes, Domingo et al., 2018; Johnsrude et al., 2013; Kreitewolf et al., 2017; Levi, 2015; Levi et al., 2011; Magnuson et al., 1995; Newman & Evers, 2007; Nygaard et al., 1994; Nygaard & Pisoni, 1998; Souza et al., 2013; Yonan & Sommers, 2000; Zhu & Holmes, 2025). Yet, whether voice familiarity also reduces effort when people try to understand speech in competing speech has not previously been examined.

It is well-documented that familiar voices are more intelligible than unfamiliar voices in noisy environments: Words spoken by friends (Domingo et al., 2019, 2020; Holmes & Johnsrude, 2020, 2021, 2023; Holmes, Domingo et al., 2018), spouses (Domingo et al., 2020; Johnsrude et al., 2013; Souza et al., 2013), family members (Barker & Newman, 2004; Magnuson et al., 1995), and personally familiar university professors (Buntrock et al., 2021; Newman & Evers, 2007) are reported more accurately than words spoken by unfamiliar people. This finding holds when voices are counterbalanced across familiar and unfamiliar conditions, such that one participant's familiar voice is another participant's unfamiliar voice (e.g., Buntrock et al., 2021; Domingo et al., 2019, 2020; Holmes et al., 2021; Holmes & Johnsrude, 2020, 2021, 2023; Holmes, Domingo et al., 2018; Johnsrude et al., 2013)—meaning that the intelligibility benefit can be ascribed to familiarity, rather than voice-specific effects. Interestingly, participants seem to gain a similar-magnitude intelligibility benefit—of approximately 10–15% improvement in sentence report—from a friend they have known for at least 6 months compared to a spouse they have cohabited with for 5 years or longer (Domingo et al., 2019). This finding implies that the familiar-voice benefit to intelligibility develops relatively quickly as people get to know each other naturally, then remains relatively stable across longer durations of time.

Studies that have trained participants to become familiar with new voices in the lab demonstrate that the familiar-voice benefit can occur even with relatively short, artificial exposure (Case et al., 2018; Holmes et al., 2021; Kreitewolf et al., 2017; Levi, 2015; Levi et al., 2011; Nygaard et al., 1994; Nygaard & Pisoni, 1998; Yonan & Sommers, 2000; Zhu & Holmes, 2025). Holmes et al. (2021) compared intelligibility for three trained voices, to which participants had been exposed for different lengths of time. They first familiarized participants with the names of three new talkers, then trained participants to recognize the names of the talkers, providing feedback on each trial. Each participant was exposed to one voice speaking 88 sentences during familiarization and training, another speaking 166 sentences, and a third speaking 478 sentences. Participants then underwent a speech intelligibility test, in which they were asked to report sentences from trained and novel voices when a competing talker was present. The results showed that the three trained voices were all more intelligible than novel voices. The voice that was trained for the longest duration of time was the most intelligible, producing a 10–15% improvement in sentence report when compared to novel unfamiliar voices—which is comparable to the magnitude of the benefit that has been reported for naturally familiar voices (Domingo et al., 2020; Holmes & Johnsrude, 2021, 2023; Holmes, Domingo et al., 2018; Johnsrude et al., 2013). Thus, training participants to recognize a voice has great potential for improving the intelligibility of those voices in noisy, everyday environments.

Understanding speech in noisy environments is particularly challenging for people with hearing loss (Gatehouse & Noble, 2004), and it has recently been recognized that listeners who achieve the same level of intelligibility might exert different amounts of effort to achieve it (McGarrigle et al., 2014). Listening effort has been defined as the “deliberate allocation of mental resources to overcome obstacles in goal pursuit when carrying out a listening task” (“Framework for Understanding Effortful Listening”; Pichora-Fuller et al., 2016). Exerting a high level of effort throughout the day can lead to excessive fatigue that particularly affects people with hearing loss (Alhanbali et al., 2017; Hornsby et al., 2016). Therefore, identifying ways to alleviate listening effort in noisy environments could help to mitigate this challenge. Potentially, voice familiarity could reduce effort as well as improving intelligibility—for example, by allowing listeners to better resist interference from competing speech (Holmes & Johnsrude, 2020), thereby decreasing cognitive load. Although, how voice familiarity affects effort in noisy environments has not previously been tested.

Studies measuring listening effort have commonly focussed on how acoustic factors contribute to effort. Measures of listening effort include a listener's subjective, self-reported effort score (Alhanbali et al., 2017; Holmes, Folkeard et al., 2018), and physiological measures, such as pupillometry (Winn et al., 2015; Zekveld et al., 2011) and electroencephalography (EEG; e.g., Dimitrijevic et al., 2019; Obleser et al., 2012), which have been suggested as objective markers of effort. These self-reported and physiological measures of listening effort do not always fully align with one another (e.g., Alhanbali et al., 2019; Shields et al., 2023). For example, Alhanbali et al. (2019) took a variety of measures while participants listened to sentences in noise, including self-reported fatigue on a visual analog scale, pupil size, and alpha power during speech perception measured with EEG. They found that each of these measures showed good internal reliability (intraclass correlation coefficients > .70), but they were only weakly correlated with one another, and loaded onto different factors in a factor analysis. Nevertheless, studies examining listening effort using various methods have consistently reported that acoustically degrading speech (e.g., Obleser et al., 2012; Winn et al., 2015) and decreasing the signal-to-noise ratio between speech and competing sounds (e.g., Wendt et al., 2016; Wiggins et al., 2025; Zekveld et al., 2011, 2014) increase effort, when tested across conditions in which the stimuli are at least partially intelligible.

Fewer studies have examined how nonacoustic factors contribute to effort. Nevertheless, Borghini and Hazan (2018) demonstrated that language proficiency contributes to effort when participants try to understand speech in background noise: They found lower pupil dilation (indicating less effort) when speech was in a listener's native language compared to when it was in a non-native language. Similarly Brown et al. (2020) found lower pupil dilation for native accents compared to unfamiliar, non-native accents. In addition, Koelewijn et al. (2015) examined how the certainty of a target talker's location affects self-reported effort ratings and pupillometry measures. Their results showed lower pupil dilation and lower self-reported effort when talker location was fixed than variable, implying that predictability affects listening effort. Semantic context can also reduce listening effort, which has been demonstrated by both lower self-reported effort (Holmes, Folkeard et al., 2018) and lower pupil dilation (Winn, 2016) for conditions with greater predictability. Together, these findings suggest that nonacoustic factors can contribute to the amount of effort that listeners exert to understand speech.

While voice familiarity has not previously been tested as a way to reduce effort when listeners try to understand speech in noisy environments, one previous study (Biçer et al., 2023) examined how voice familiarity affects effort during a voice-cue discrimination task. In this task, participants were asked to determine which of three stimuli was the odd-one-out, based on differences in the fundamental frequency and vocal tract length of the voice. They assessed listening effort during this discrimination task using pupillometry. Biçer et al. (2023) examined how implicit training with a new voice affected voice-cue discrimination, by exposing participants to a new voice through passive listening, and comparing voice-cue discrimination for the trained voice with novel voices. Their results showed no differences in the accuracy of voice-cue discrimination between the trained and unfamiliar voices. Although they found some evidence of lower pupil dilation for familiar voices. Thus, these results imply that voice familiarity has potential to reduce effort, but this has never been studied during a speech intelligibility task.

Here, we aimed to examine how voice familiarity affects the effort that listeners exert during speech perception when competing speech is present, alongside improvements in performance. Similar to Holmes et al. (2021), we explicitly trained participants to become familiar with three new voices for different durations of time (speaking 88, 166, or 478 sentences during familiarization and training), so that we could study performance and effort during the initial stages of voice learning. We compared listening effort for trained and unfamiliar voices during speech-in-speech perception, using both self-reported effort and pupillometry. We hypothesized that trained voices would be associated with better performance, lower self-reported effort, and lower pupil dilation, compared to unfamiliar voices. In addition, we hypothesized that we might find differences among the three trained voices, with longer training leading to better performance and lower effort.

## Methods

### Transparency and Openness

The stimuli (https://osf.io/xajkq/?view_only=0c3cef41e75e4c67bd16c32549e08dc3), data (https://osf.io/b8gez/?view_only=2031708b23214165b08aea84ed108042), and analysis scripts (https://osf.io/b8gez/?view_only=2031708b23214165b08a.ea84ed108042) are available on the Open Science Framework. The design and its analyses were not preregistered.

### Participants

We did not know in advance the sizes of expected voice familiarity effects on self-reported effort or pupil dilation. Therefore, we targeted a final sample size of 20, because this sample size was used by Winn (2016) to examine how pupil dilation is affected by semantic context in participants with normal hearing.

To examine the sensitivity of a sample size of 20, we conducted power analyses using G*Power (version 3.1.9.2; Faul et al., 2007) with an alpha of .05. When using a two-tailed *t*-test to test a difference between two dependent means (such as a comparison between a familiar and unfamiliar voice), 20 participants provides 80% power to detect effect sizes of *d_z_* ≥ .66 (i.e., moderate-sized effects). Familiar-voice effects on *speech intelligibility* have been reported to be much larger than this: for example, the familiar-voice intelligibility benefit had a size of *d_z_* = .94 in Holmes et al. (2021), and effects of *d_z_* = .94 are detectable with 98% power in a sample size of 20 (using a *t*-test to detect a difference between two dependent means). We thought it was plausible that effects of voice familiarity on self-reported effort and pupil dilation could be smaller than effects on intelligibility, but we anticipated that 20 participants would be an appropriate sample size, given our expectation of medium-to-large effect sizes.

As planned, we analyzed data from 20 participants. We recruited two additional participants who did not complete the study because the eye-tracker calibration failed. Participants were aged 18–34 years (median = 23.5 years, interquartile range = 5.5). Ten were male, nine were female, and one identified as nonbinary. All participants were native English speakers and had normal or corrected-to-normal vision. Two participants wore glasses throughout the experiment and one participant wore contact lenses. Participants reported no history of neurological issues, hearing loss, or hearing difficulties. They had average pure-tone thresholds (measured at octave frequencies between 5 and 8 kHz using a Resonance R07A Portable Screening Audiometer) better than 15 dB HL in each ear (mean = 3.3 dB HL, standard deviation = 3.3). Participants were instructed not to consume caffeine for a period of 6 h before starting the study, because caffeine can affect pupil responses (Winn et al., 2018).

The study gained ethical approval from the UCL Psychology and Language Sciences Local Research Ethics Committee. Participants provided written informed consent and were compensated for their time.

### Design

**Figure 1.**
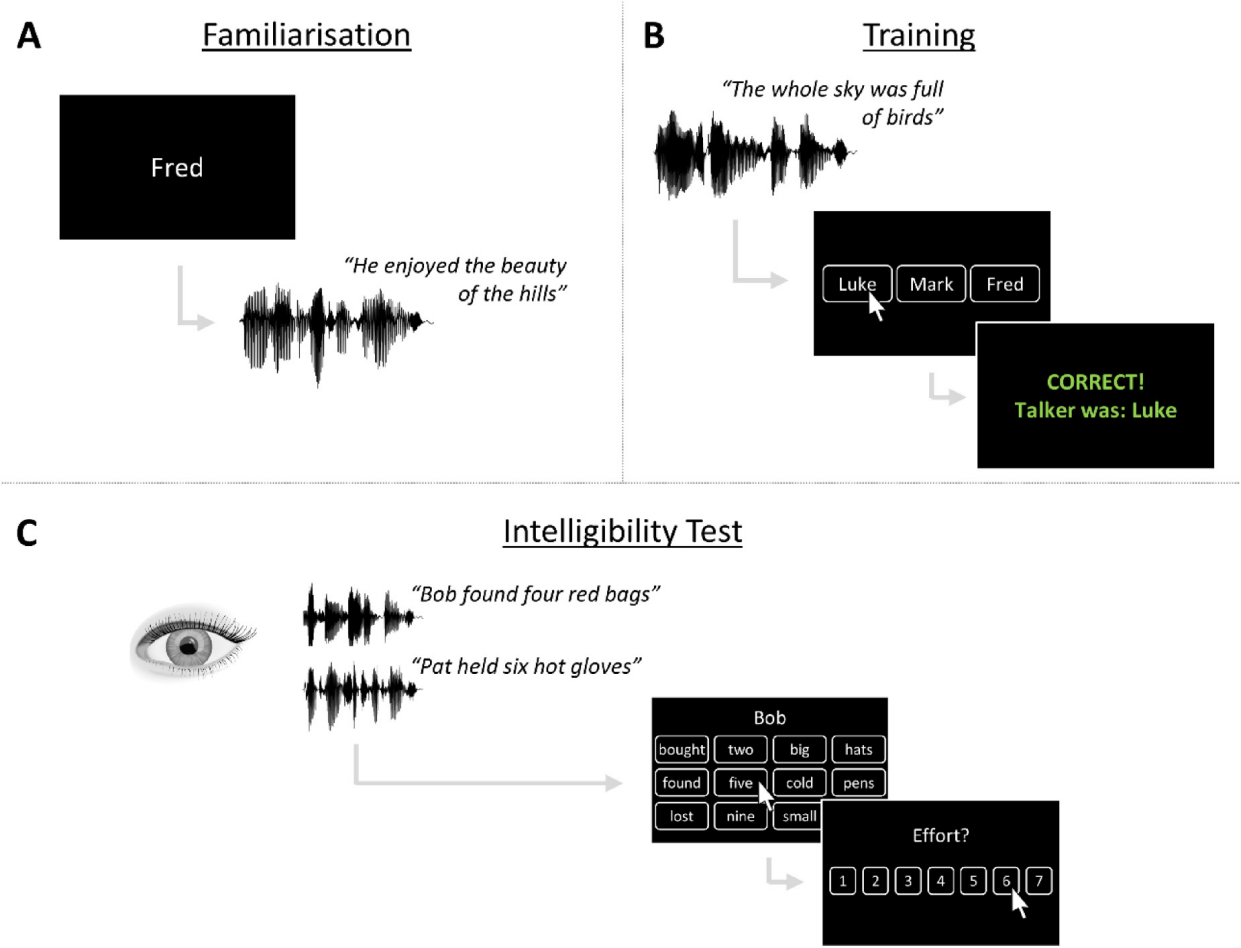
Schematics of the three phases of the study: (A) Familiarization, (B) training, and (C) speech-intelligibility test. Note that the first response screen for the intelligibility test contains only three rows of words for clarity, but eight rows were shown (i.e., displaying all word options) during the study.

The study involved three main phases: (i) familiarization with three novel voices (10 sentences for each voice), (ii) voice-identification training with the same three voices (“Least Familiar”: 78 sentences; “Moderately Familiar”: 156 sentences; “Most Familiar”: 468 sentences), and (iii) a speech-intelligibility test containing the three trained voices and two novel voices (Figure 1). The familiarization and training phases exposed participants to the same number of sentences as in Holmes et al. (2021)—for a total of 88, 166, and 478 sentences. Across participants, the assignment of the five voices to the familiar and unfamiliar conditions was counterbalanced, as was the assignment of sentences to voices. The study lasted approximately 3 h in total, including breaks. Participants were encouraged to take self-paced breaks between tasks and also between blocks of trials within each task.

In the intelligibility test, participants reported words from a target sentence in the presence of a competing sentence spoken by a different talker. Two factors were manipulated in the intelligibility test: the familiarity of the voice that spoke the target sentence (Most Familiar, Moderately Familiar, Least Familiar, or Unfamiliar), and the target-to-masker ratio (TMR). As in previous voice familiarity studies (Holmes et al., 2021; Holmes, Domingo et al., 2018; Zhu & Holmes, 2025), the TMR for each trial was either −6 or +3 dB, reflecting different levels of acoustic challenge. Based on previous studies (Domingo et al., 2020; Holmes et al., 2021; Holmes & Johnsrude, 2023; Holmes, Domingo et al., 2018), −6 and +3 dB TMR avoid ceiling and floor effects; in addition, having one positive and one negative TMR means that participants cannot use TMR as a cue to determine the target sentence.

### Apparatus

The acoustic stimuli were recorded in a sound-attenuating booth (IAC Acoustics UK Ltd), using a RØDE NT1-A microphone (The Freedman Group, Sydney) connected to a FireFace UC sound card (RME Audio Interfaces, Germany).

The experiment took place in a double-walled audiometric booth (120a Series, IAC Acoustics UK Ltd). Acoustic stimuli were delivered diotically through Sennheiser HD200 pro headphones. Participants were seated in a comfortable, adjustable chair, facing a Dell U2715H LCD monitor.

During the speech-intelligibility test, pupillometry was conducted with an Eyelink 1000 Plus (SR Research Ltd) eye-tracker, and participants were required to use a headrest. The headrest was positioned for each participant so that looking at the entire screen was comfortable. The illumination inside the audiometric booth was kept constant across participants.

The left eye was selected as the tracked eye for all participants. The pupil and corneal reflection thresholds were set manually for each participant, ensuring that no shadows, noise, or reflections were erroneously detected. The eye-tracker was calibrated for each participant at the beginning of the experiment using a 9-point sequence. Gaze fixations were manually accepted when they were stable. The calibrations were then validated. The calibration was only accepted when the maximum degree of spatial error during validation was less than .5 degrees. Otherwise, we repeated the calibration process until this criterion was met. The eye-tracker was set to measure pupil diameter. Recordings were made at either 1,000 Hz (*N* = 16) or 500 Hz (*N* = 4).

### Stimuli

The sentences were the same as those used in Holmes et al. (2021). Different sets of sentences were used for the familiarization and training phases compared to the test phase. For the familiarization and training phases, we used 354 meaningful sentences based on the sentence corpus developed by Rodd et al. (2005); for example, “The daisies began to grow quite soon.” For the speech-intelligibility test, sentences were taken from the Boston University Gerald (BUG) corpus (Kidd et al., 2008). Sentences from the BUG corpus each have five words, which follow the structure, “*Name verb number adjective noun*”; for example, “Pat lost four red bags.” The name was always fixed as either “Bob” or “Pat,” and each of the four remaining words had eight possible options (see Table 1). We selected 384 sentences from this corpus for the speech-intelligibility test. An advantage of using these closed-set matrix sentences for the speech-intelligibility test, rather than open-set sentences, is that participants are unable to guess the correct response based on semantic probability or the absence of semantic neighbors. In addition, the test requires participants to make four separate and unrelated responses, and to guess if uncertain, which eliminates the possibility that familiar voices are more intelligible because participants are more willing to guess words for familiar voices than for unfamiliar voices (i.e., a change in criterion or bias). In contrast, open-set tests that ask participants to report as many target words as they heard could result in different estimates of performance between familiar and unfamiliar voices, regardless of sensitivity, if participants feel less confident reporting words for unfamiliar voices, so report fewer words overall.

**Table 1.** Words from the BUG Corpus That Were Used in the Speech-Intelligibility Test.

Name	Verb	Number	Adjective	Noun
Bob	bought	two	big	bags
Pat	found	three	blue	cards
	gave	four	cold	gloves
	held	five	hot	hats
	lost	six	new	pens
	saw	eight	old	shoes
	sold	nine	red	socks
	took	ten	small	toys

Each sentence contained five words: one name, verb, number, adjective, and noun.

The sentences were recorded by five male, native English speakers aged 22–37 years (median *=* 31 years, interquartile range = 12). While recording the sentences, the speakers were encouraged to use their natural speaking style, while keeping to a desired pace that was indicated by a video for each sentence (Holmes, 2018). The sentences were recorded in stereo at a sample rate of 44100 Hz, then converted to mono. After conversion, sentences were normalized to the same root-mean-square amplitude.

### Procedure

During the familiarization phase, participants were presented with 30 sentences spoken in three voices. We presented 10 sentences for each voice, which were randomly interleaved across trials. While each sentence was playing, a name was displayed on the screen, which participants were told to associate with the voice (Figure 1A). The three names were “Mark,” “Fred,” and “Luke,” which were pseudo-randomly paired with the three voices and counterbalanced across participants.

During the training phase, participants heard sentences from the same three voices as the familiarization phase. On each trial, participants heard a sentence and saw the three name options on the screen (Figure 1B). They were asked to identify the name that corresponded to the voice. They could respond at any time after the sentence had begun; although, the sentence always played in full, even if participants responded before the end of the sentence. This ensured that all participants received the same duration of training across the three voices. After the sentence had finished, participants received feedback about their response (“Correct” displayed in green font or “Incorrect” displayed in red font) and were presented with the correct name (e.g., “Talker was Fred”) regardless of whether they correctly identified the name. Of the three voices, one spoke 78 sentences (“Least Familiar”), another spoke 156 sentences (“Moderately Familiar”), and the third spoke 468 sentences (“Most Familiar”), corresponding to approximately 10, 20, and 60 min of training. The talker was selected pseudo-randomly on each trial. During the training phase, 351 sentences were used, which were each presented twice across the training phase, but in a different voice each time they were presented.

Finally, participants completed the speech-intelligibility test (Figure 1C). On each trial, participants heard two BUG sentences simultaneously. One sentence started with “Bob” and the other started with “Pat.” The target sentence was defined by the first word of the sentence, which was “Bob” for one half of the test and “Pat” for the other half of the test, the order of which was counterbalanced across participants. The participant was reminded of the target word at the beginning of the speech-intelligibility test and at the half-way point, when the target word switched. The target voice could be one of the three trained (“familiar”) voices or one of two novel (“unfamiliar”) voices that participants had not heard during training. The masker voice was always one of the two novel voices: when the target was a trained voice, the masker was one novel voice for half of trials, and the other novel voice for the other half of trials (pseudo-randomly selected); when the target was a novel voice, the masker was the other novel voice. This gave rise to four different target-familiarity conditions: Unfamiliar, Least Familiar, Moderately Familiar, and Most Familiar. In total, the speech-intelligibility test contained 192 trials: 48 for each familiarity condition. Within each familiarity condition, the stimuli were presented equally often (24 trials each) at +3 dB and −6 dB TMR.

**Figure 2.**
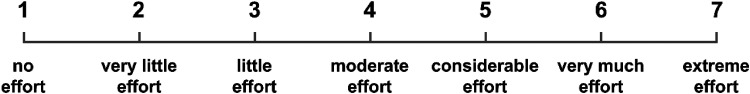
Visual depiction of the scale used for participants’ self-reported effort scores.

Participants’ pupil responses were recorded throughout the speech-intelligibility test. At the start of each trial of the speech-intelligibility test, a fixation cross was presented for 1 s to stabilize the participant's gaze and establish a reliable baseline period. Next, the acoustic stimuli began while the fixation cross remained on the screen. The acoustic stimuli lasted approximately 1.5 s, and the first response screen appeared 2 s after the offset of the acoustic stimuli. This delay was implemented to separate pupil responses to acoustic stimuli from participants’ responses. Participants were instructed to report the four words from the target sentence (which began with the target word, “Bob” or “Pat”) by choosing a word from each of four columns that were presented on the screen (Figure 1C). Each column contained eight words. Participants were required to select the four words from the target sentence by clicking one word from each column, in any order. After the participant had reported the words from the target sentence, they were presented with another response screen, which asked them to report the amount of effort that they had exerted during that trial. The scale ranged from 1 to 7, where 1 denoted “no effort” and 7 denoted “extreme effort” (as in Holmes, Folkeard et al., 2018). On each trial, participants were required to select a number from 1 to 7 (without effort labels). At the beginning of the speech-intelligibility test, the experimenter gave participants a handout that visually depicted the scale and its associated labels, which is shown in Figure 2. In addition, the handout encouraged participants to use the full scale and explained the distinction between effort and difficulty (see Supplemental Material). Participants were encouraged to ask the experimenter questions about the effort scale, to ensure appropriate self-report. Participants were also given guidance about how to best-time their blinks during the test, with the aim of obtaining clean pupil data. There was a variable intertrial interval of 3.5–4.5 s.

Prior to the speech-intelligibility test, participants completed a practice session containing eight trials. The practice trials were all presented at a TMR of 0 dB, to ensure that participants learnt to identify the target sentence based on its first word, rather than using the level of the sentence to distinguish the target and masker sentences. Otherwise, practice trials were identical to those in the speech-intelligibility test.

### Preprocessing

The EyeLink files were converted with the Edf2Mat MATLAB Toolbox (designed and developed by Adrian Etter and Marc Biedermann at the University of Zurich), then were preprocessed using the Pupillometry Pipeliner toolbox (Pupl; Kinley & Levy, 2022) in MATLAB (version 2024b). Pupil data that were recorded at a sample rate of 1,000 Hz (*N* = 16) were first downsampled to 500 Hz, so that all data had a sample rate of 500 Hz. Blinks were identified using the pupillometry noise method (Hershman et al., 2018). Each blink, and a 50-ms window either side of the blink, was removed from the data. We then applied linear interpolation (using a maximum duration of 500 ms and a maximum gap of 1 standard deviation) to reduce the amount of missing data. To deal with high-frequency artifacts, we applied a Hann window moving average filter (with a width of 150 ms) to the continuous data before epoching. We applied baseline correction to the epoched data by subtracting the mean pupil diameter across the 1,000-ms prestimulus baseline period for each trial (corresponding to the time during which the fixation cross was displayed). Epochs were rejected if the proportion of missing data exceeded 18% (Lemercier et al., 2014). On average, 9.74% of epochs were rejected per participant. Epochs were separated into 8 conditions (4 target-familiarity conditions × 2 TMR conditions).

**Figure 3.**
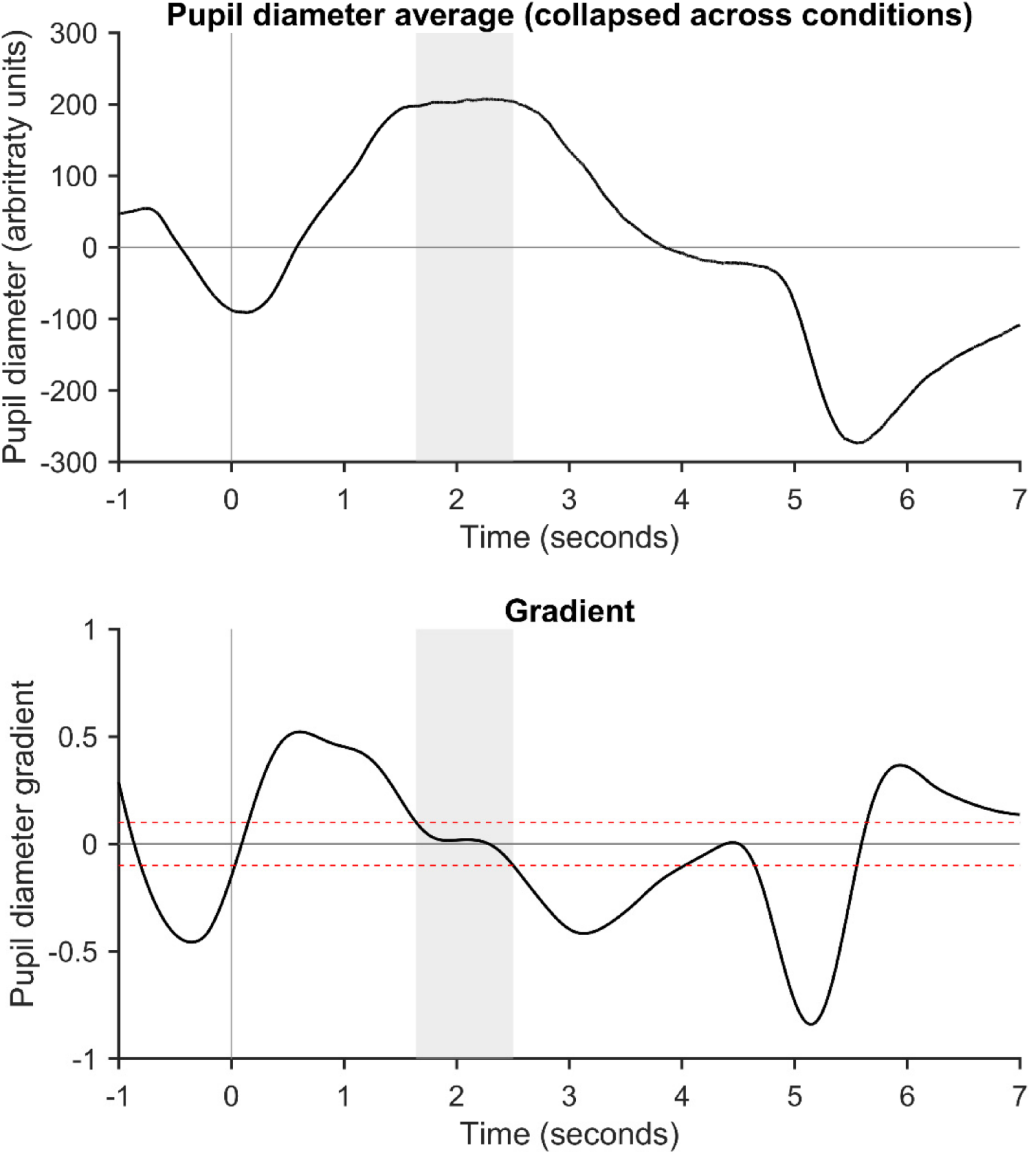
Upper panel: Grand average pupil diameter (*N* = 20), collapsed across target familiarity and TMR conditions. Only Unrejected epochs are included in the average. Lower panel: Gradient of the pupil diameter time course. Red horizontal lines show ± .1, which were the boundaries used to identify parts of the time course across which there was little change in pupil diameter. The peak time window was defined as the period where pupil diameter was greatest (upper panel) and the gradient was within ± .1 (lower panel). The gray shaded area shows the selected peak time window, which was used for subsequent statistical analyses.

To compare pupil dilation across conditions, we calculated the mean pupil diameter across trials, which has been commonly used as a dependent variable in previous pupillometry studies (Kinley & Levy, 2022; Kret & Sjak-Shie, 2019; Winn et al., 2018). We aimed to measure pupil diameter over the portion of the trial for which pupil dilation was maximal, although we wanted to avoid picking a single peak value, given that the exact peak is highly sensitive to noise. To this end, we defined a time window around the peak using a novel method. Specifically, we looked at the gradient of the average pupil response (i.e., the change in pupil diameter) across all epochs, averaged across participants (Figure 3). We defined the maximal pupil dilation time window as the time during the trial at which pupil diameter was at its greatest, and during which the gradient (i.e., the change in diameter) was within ± .1. Under this calculation, the difference in pupil diameter between adjacent samples was < .1. This provided a time window for pupil analysis at 1.6407–2.5011 s after the onset of acoustic stimuli. We calculated the mean pupil diameter for each condition across this time window.

### Analyses

We conducted analyses using RStudio (version 2024.12.1; Build 563) with R version 4.5.0.

To evaluate accuracy during the training phase, we calculated the proportion of trials for which participants correctly identified the voice. Shapiro–Wilk tests showed that the training data were not normally distributed (*p* ≥ .027), so we used a Friedman rank sum test to compare accuracy across the three voices that were trained for different lengths of time (Least Familiar, Moderately Familiar, and Most Familiar). We used Wilcoxon signed rank tests to compare pairs of adjacent Familiarity conditions (i.e., Least Familiar compared with Moderately Familiar, and Moderately Familiar compared with Most Familiar). To carry out these analyses, we used the friedman.test() and wilcox.test() functions from the R “stats” package.

For the speech-intelligibility test, we were interested in behavioral and pupillometry measures. We calculated speech intelligibility as the percentage of trials for which participants reported all four words from the target sentence correctly. We calculated self-reported effort as the mean of participants’ effort ratings across trials. For pupil dilation, we calculated the mean pupil diameter across the 1.6407–2.5011 s time window. None of the data from the speech-intelligibility test violated normality assumptions, as assessed by Shapiro–Wilk tests and visual inspection of the data. We evaluated the effects of Target Familiarity (Unfamiliar, Least Familiar, Moderately Familiar, Most Familiar) and TMR (−6 dB, + 3 dB) on each measure using two-way repeated-measures ANOVAs. We ran five planned contrasts, to compare each of the familiar-target conditions (Least Familiar, Moderately Familiar, and Most Familiar) with the unfamiliar condition, and to compare pairs of adjacent familiar-target conditions (i.e., Least Familiar compared with Moderately Familiar, and Moderately Familiar compared with Most Familiar). We used the aov_ez() function from the R “afex” package for the ANOVAs, and we used the emmeans() and contrast() functions from the R “emmeans” package for planned contrasts. Where Mauchley's test of sphericity gave a significant result, we report Greenhouse–Geisser corrected *p*-values.

To examine whether self-report and pupillometry measures of effort were differentially sensitive to effects of TMR and Target Familiarity, we converted the mean effort ratings and mean pupil diameter for each participant into z-scores, and entered them into a three-way repeated-measures ANOVA. The three factors were Measurement (Self-report or Pupil), Target Familiarity, and TMR. For this analysis, we were primarily interested in interactions with Measurement.

## Results

### Training

**Figure 4.**
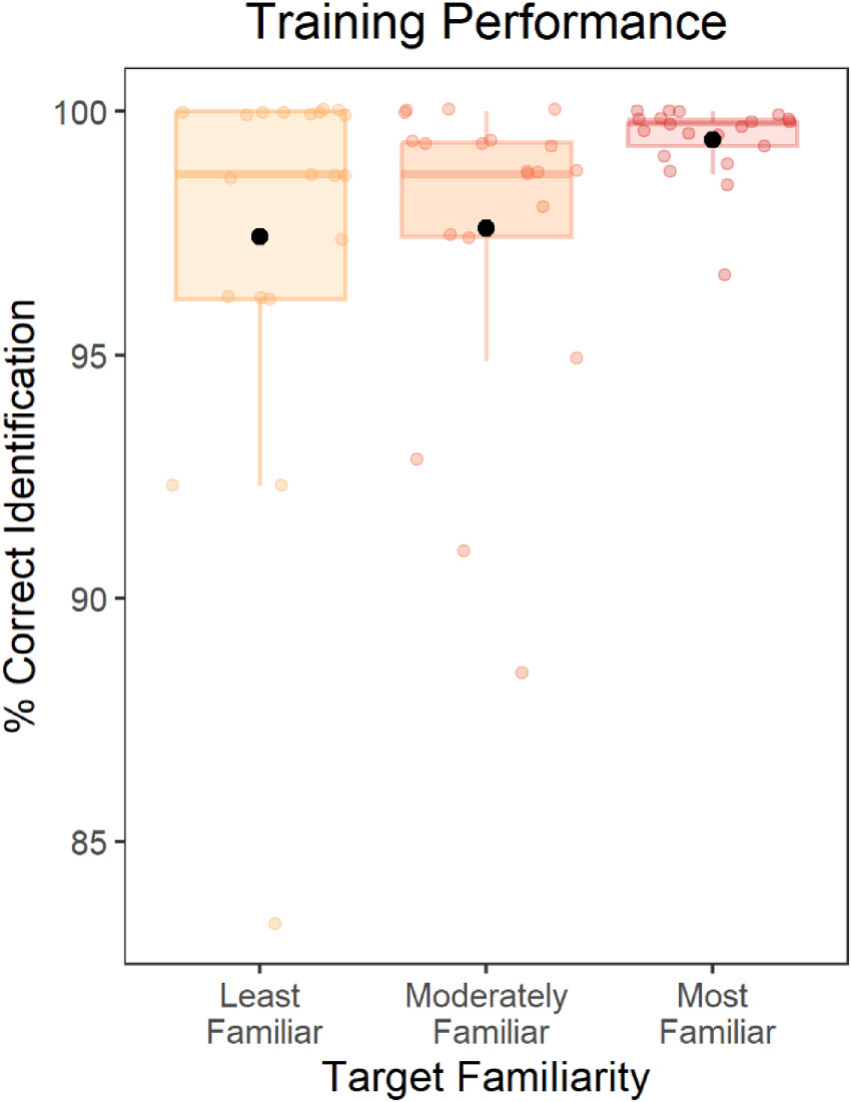
Accuracy of voice identification for the three talkers in the training phase. Each box displays the interquartile range, and the thick horizontal lines display the medians. Colored dots illustrate data from individual participants, and the black dots illustrate the means across participants.

Accuracy in the training phase was high overall (Figure 4) and participants performed close to ceiling. Numerically, accuracy was highest for the Most Familiar voice (mean = .99, standard deviation [sd] = .08), followed by the Moderately Familiar voice (mean = .98, sd = .15), and was lowest for the Least Familiar voice (mean = .97, sd = .16). A Friedman rank sum test showed a significant effect of familiarity on accuracy, χ^2^(2) = 6.77, *p* = .034. Wilcoxon signed rank tests showed a significant difference between the Most Familiar and Moderately Familiar conditions (*V* = 13.0, *p* = .001), and no significant difference between the moderately familiar and least familiar conditions (*V* = 74.5, *p* = .65). Thus, accuracy was high for all three trained voices, but was better for the Most Familiar voice than for the two voices that participants heard for shorter durations during training.

### Speech-Intelligibility Test: Accuracy

**Figure 5.**
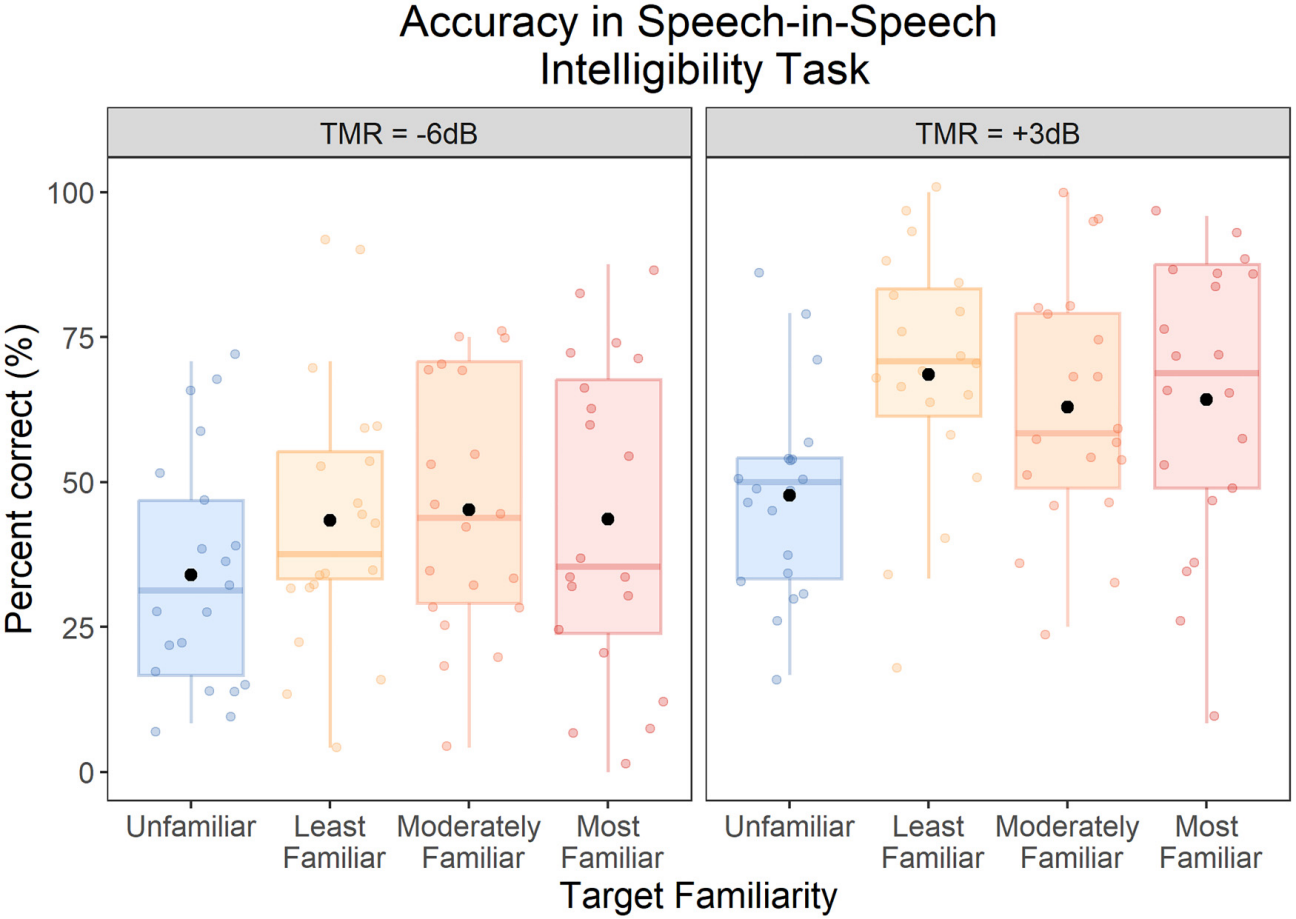
Accuracy for the speech-intelligibility test, by target familiarity and TMR conditions. Each box displays the interquartile range, and the thick horizontal lines display the medians. Colored dots illustrate data from individual participants, and the black dots illustrate the means across participants.

Figure 5 displays the accuracy results. We found a significant main effect of TMR, *F*(1, 19) = 81.17, *p* < .001, *η*_p_^2^ = .81, with better performance at +3 than −6 dB TMR. We also found a significant main effect of Target Familiarity, *F*(3, 57) = 4.79, *p* = .005, *η*_p_^2^ = .20. Planned comparisons, comparing each of the familiar conditions with the unfamiliar conditions, revealed a significant familiar-voice benefit to intelligibility when the target was the Least Familiar voice (mean benefit of 15.1%, SE = 4.45), *t*(19) = 3.39, *p* = .003, *d_z_* = .76, 95% CI: [.25, 1.25], the Moderately Familiar voice (mean benefit of 13.2%, SE = 4.69), *t*(19) = 2.82, *p* = .011, *d_z_* = .63, 95% CI: [.14, 1.11], and the Most Familiar voice (mean benefit of 13.0%, SE = 4.64), *t*(19) = 2.80, *p* = .011, *d_z_* = .63, 95% CI: [.14, 1.10]. There were no significant differences between the Least Familiar and Moderately Familiar conditions, *t*(19) = .41, *p* = .69, *d_z_* = .09, 95% CI: [−.35, .53], or between the Moderately Familiar and Most Familiar conditions *t*(19) = .05, *p* = .96, *d_z_* = .01, 95% CI: [−.43, .45].

There was a significant interaction between familiarity and TMR, *F*(3, 57) = 3.28, *p* = .027, *η*_p_^2^ = .15. To examine this interaction, we conducted a one-way ANOVA to examine the effect of Target Familiarity at each TMR. We found a significant effect of Target Familiarity at +3 dB TMR, *F*(3, 57) = 7.41, *p* < .001, *η*_p_^2^ = .28, but not at −6 dB TMR, *F*(3, 57) = 2.06, *p* = .12, *η*_p_^2^ = .10. Thus, the effect of familiarity was confined to the more favorable TMR condition.

To follow-up on the significant difference among Target Familiarity conditions at +3 dB TMR, we ran post hoc tests with Bonferroni correction. All three familiar conditions differed from the unfamiliar condition [Most Familiar: *t*(19) = 3.38, *p* = .016, *d_z_* = .76, 95% CI: .25, 1.25; Moderately Familiar: *t*(19) = 3.17, *p* = .025, *d_z_* = .71, 95% CI: .21, 1.19; Least Familiar: *t*(19) = 4.30, *p* = .002, *d_z_* = .96, 95% CI: .42, 1.49]. There were no significant differences between the Least Familiar and Moderately Familiar conditions, *t*(19) = 1.27, *p* = 1.00, *d_z_* = .28, 95% CI: [−.17, .73], or between the Moderately Familiar and Most Familiar conditions *t*(19) = .28, *p* = 1.00, *d_z_* = .06, 95% CI: [−.38, .50].

### Speech-Intelligibility Test: Self-Reported Effort

**Figure 6.**
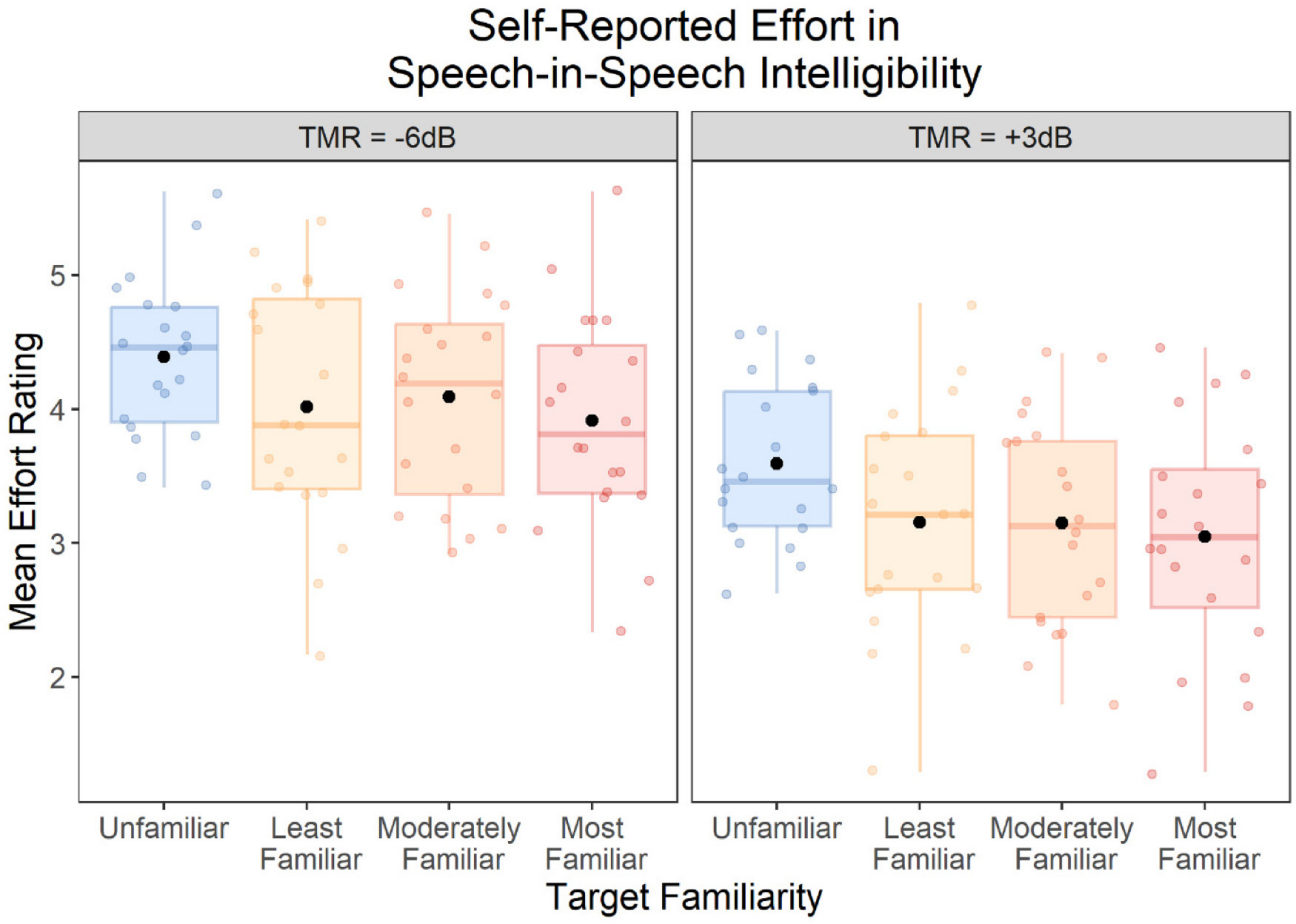
Self-reported effort scores (possible range: 1–7) on the speech-intelligibility test, by target familiarity and TMR. Each box displays the interquartile range, and the thick horizontal lines display the medians. Colored dots illustrate data from individual participants, and the black dots illustrate the means across participants.

Self-reported effort ratings during the speech-intelligibility test are displayed in Figure 6. We found a significant main effect of TMR, *F*(1, 19) = 62.01, *p* < .001, *η*_p_^2^ = .77, with lower self-reported effort at +3 than −6 dB TMR. There was also a significant main effect of Target Familiarity, *F*(3, 57) = 3.88, *p* = .014, *η*_p_^2^ = .17. Planned contrasts revealed significantly less self-reported effort in all three familiar-target conditions when compared to the Unfamiliar condition [Least Familiar: *t*(19) = 2.78, *p* = .012, *d_z_* = .62, 95% CI: [.13, 1.09]; Moderately Familiar: *t*(19) = 2.34, *p* = .030, *d_z_* = .52, 95% CI: [.05, .99], and Most Familiar: *t*(19) = 2.93, *p* = .009, *d_z_* = .66, 95% CI: [.16, 1.13]]. However, there were no significant differences between the Least Familiar and Moderately Familiar conditions, *t*(19) = .25, *p* = .81, *d_z_* = .05, 95% CI: [−.38, .49], or between the Moderately Familiar and Most Familiar conditions *t*(19) = .90, *p* = .38, *d_z_* = .20, 95% CI: [−.24, .64].

There was no significant interaction between TMR and Target Familiarity, *F*(3, 57) = .29, *p* = .83, *η*_p_^2^ = .02, indicating that the effect of Target Familiarity on self-reported effort did not differ between the two TMR conditions.

### Speech-Intelligibility Test: Pupillometry

**Figure 7.**
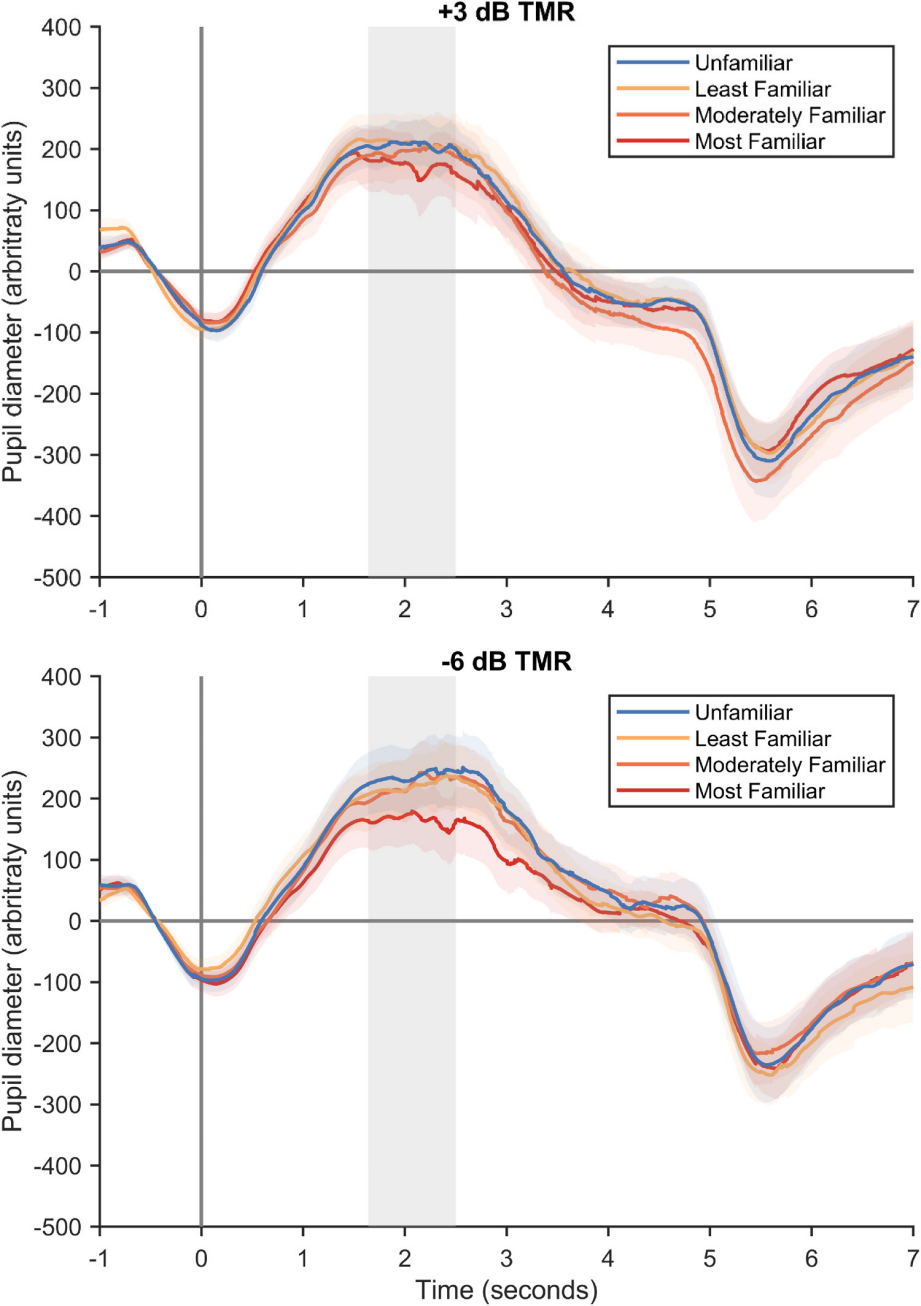
Grand average pupil diameter (*N* = 20) across target familiarity and TMR (+3 and −6 dB) conditions. Only unrejected epochs are included in the averages. Error bars show the standard error of the mean. Gray shaded areas indicate the peak time window that was used for statistical analyses.

**Figure 8.**
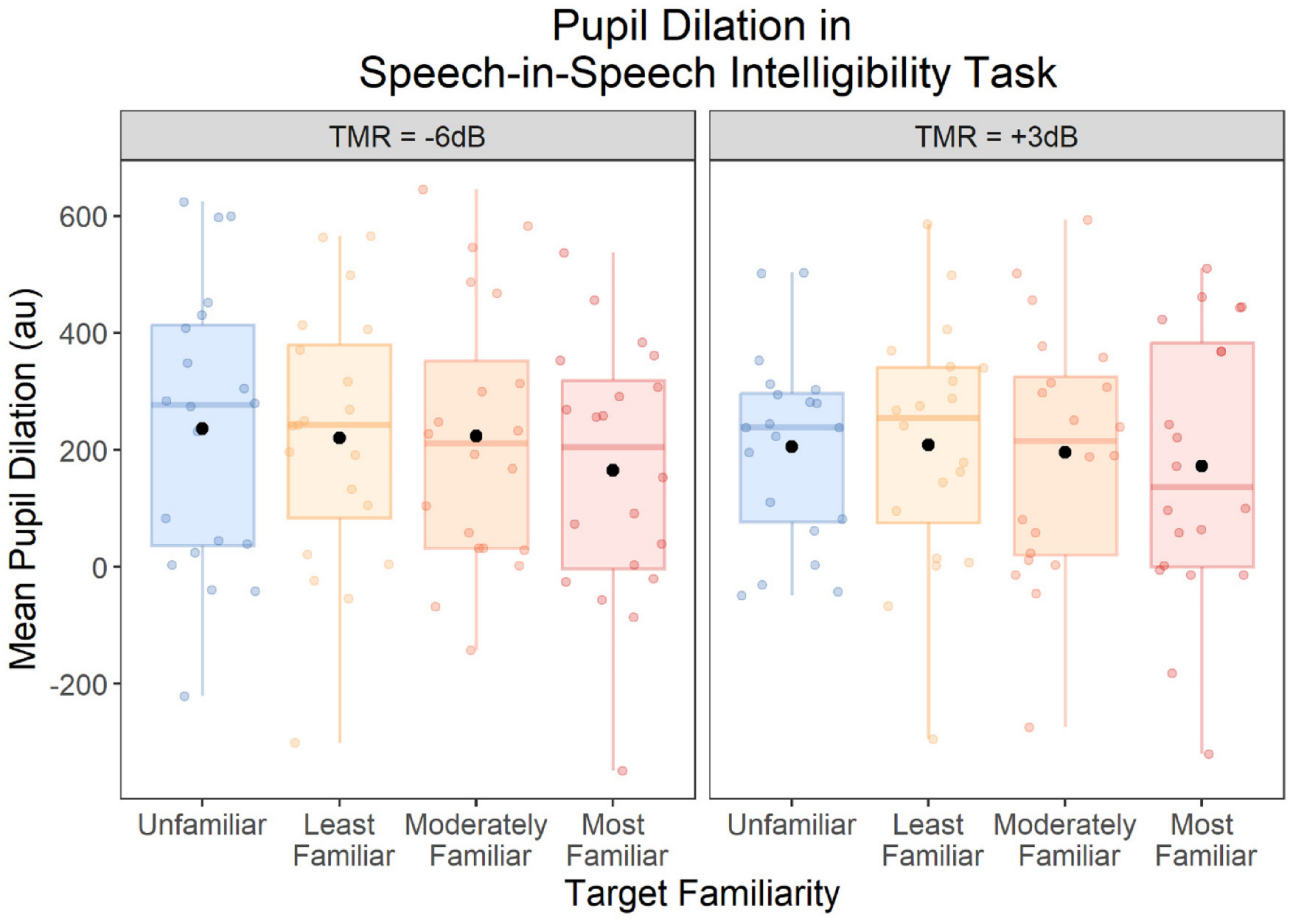
Mean pupil diameter across the peak time window (1.6407–2.5011 s) during the speech-intelligibility test. Data are plotted by target familiarity and TMR. Each box displays the interquartile range, and the thick horizontal lines display the medians. Colored dots illustrate data from individual participants, and the black dots illustrate the means across participants.

Pupil time courses, separated by TMR and Target Familiarity conditions, are displayed in Figure 7. Figure 8 shows the averages across the peak time window.

Comparing mean pupil diameter across conditions, there was a significant main effect of Target Familiarity, *F*(3, 57) = 3.47, *p* = .030, $\eta_{p}^{2}$ = .15. Planned contrasts revealed significantly smaller pupil diameter (consistent with less effort) in the Most Familiar condition than the Unfamiliar condition, *t*(19) = .2.23, *p* = .038, *d_z_* = .50, 95% CI: [.03, .96]. However, there were no significant differences in pupil diameter between the Unfamiliar condition and either the Moderately Familiar, *t*(19) = .82, *p* = .421, *d_z_* = .18, 95% CI: [−.26, .62], or the Least Familiar, *t*(19) = .43, *p* = .671, *d_z_* = .10, 95% CI: [−.34, .53], conditions. In addition, pupil diameter was significantly smaller in the Most Familiar than the Moderately Familiar condition, *t*(19) = 2.22, *p* = .039, *d_z_* = .50, 95% CI: [.02, .96], but there was no significant difference between the Moderately Familiar and Least Familiar conditions, *t*(19) = .42, *p* = .677, *d_z_* = .09, 95% CI: [−.35, .53].

The main effect of TMR on mean pupil diameter was not significant, *F*(1, 19) = 2.22, *p* = .153, $\eta_{p}^{2}$ = .11, and there was no significant interaction between TMR and Target Familiarity, *F*(3, 57) = .39, *p* = .758, $\eta_{p}^{2}$ = .02.

### Relationships among Effort Measures

When we entered both effort measures (self-reported effort and pupil diameter) into an analysis together, we found significant main effects of TMR, *F*(1, 19) = 70.2, *p* < .001, $\eta_{p}^{2}$ = .79, and Target Familiarity, *F*(3, 57) = 5.07, *p* = .003, $\eta_{p}^{2}$ = .21. There was no significant interaction between TMR and Target Familiarity, *F*(3, 57) = .26, *p* = .851, $\eta_{p}^{2}$ = .01.

Given that the results from each measure were converted to z-scores, there was no significant main effect of Measurement, *F*(1, 19) < .01, *p* = 1.0, $\eta_{p}^{2}$ = 0.0, as expected. There was no significant interaction between Measurement and Target Familiarity, *F*(3, 57) = 2.14, *p* = .106, $\eta_{p}^{2}$ = .10. However, we found a significant interaction between Measurement and TMR, *F*(1, 19) = 40.75, *p* < .001, $\eta_{p}^{2}$ = .68, showing that self-reported effort ratings were more sensitive to the difference between the two TMRs than was pupil dilation. The three-way interaction (Measurement × TMR × Target Familiarity) was not significant, *F*(3, 57) = .47, *p* = .702, $\eta_{p}^{2}$ = .02.

## Discussion

Overall, we found that training participants to become familiar with new voices improves speech intelligibility and reduces the amount of effort exerted during speech-in-speech perception. Both effort measures (self-report and pupil dilation) indicated lower effort for the Most Familiar voices (that were trained for the longest) compared to unfamiliar voices, demonstrating that ∼1 h of explicit training is sufficient to reliably reduce effort.

There are several possible reasons why voice familiarization training led to lower effort. First, a familiar talker's voice may be more predictable than an unfamiliar talker's voice, which may result in better intelligibility and lower effort. The finding that greater predictability results in lower pupil dilation has been reported for simple tone sequences (Milne et al., 2021; Qiyuan et al., 1985) and for speech stimuli (Koelewijn et al., 2015). For example, Koelewijn et al. (2015) found lower pupil dilation and lower self-reported effort when a talker's location was fixed compared to variable across trials. They also found that fixing the talker across trials reduced self-reported effort, although they found no effects of talker variability on pupil dilation. Possibly, the effect of talker familiarization that we studied here generates stronger predictions than does trial-to-trial constancy in talker identity, which could explain why we found a significant effect on pupil dilation, whereas Koelewijn et al. (2015) did not. This explanation is consistent with our finding that pupil dilation was only significantly lower for the Most Familiar voices compared to unfamiliar voices, whereas the two familiar voices to which participants were exposed for shorter lengths of time (Moderately Familiar and Least Familiar) showed no significant differences in pupil dilation when compared to unfamiliar voices.

A second possible explanation for why voice familiarization training led to lower effort is that it allows listeners to better resist interference from a competing talker, which could reduce the cognitive load of listening to speech in competing speech. Under this explanation, familiar voices do not need to be more predictable than unfamiliar voices. Instead, reduced interference could be explained by quicker or more efficient processing of speech spoken by familiar talkers, leaving more cognitive resources for processing competing speech. This explanation is more consistent with the behavioral findings of Holmes and Johnsrude (2020), who showed that the familiar-voice benefit for naturally familiar voices scales with the extent of linguistic similarity of the masker.

It is worth noting that the effect of familiarity on pupil dilation we observed here is in the opposite direction than would be expected based on recollection (e.g., Papesh et al., 2012; Võ et al., 2008) or emotional arousal (e.g., Bradley et al., 2008) accounts, which would predict *greater* pupil dilation for familiar than unfamiliar voices. For example, familiar pieces of music have been associated with greater pupil dilation than unfamiliar pieces of music during passive listening (Jagiello et al., 2019). In the current task, participants’ focus was on the content of speech spoken by familiar and unfamiliar talkers, rather than the voices themselves, which may be why we observed a different pattern of pupil responses. Given that the Most Familiar voice elicited significantly lower pupil responses than unfamiliar voices, our results are most consistent with an explanation based on reduced listening effort during challenging speech perception. This pattern of results is consistent with Biçer et al.'s (2023) finding that familiar voices are associated with lower pupil dilation during a voice-cue discrimination task. It is also consistent with findings that listening to a native accent is associated with lower pupil dilation than listening to an unfamiliar, non-native accent during speech perception (Brown et al., 2020).

The degree of familiarity appeared to have different effects on self-report and pupil dilation measures. Self-reported effort was lower for all three trained voices compared to unfamiliar voices, with no difference between the three voices that participants heard for different lengths of time during training. These findings indicate that even relatively short (∼10 min) exposure to a voice can provide measurable benefits for self-reported effort. Whereas voice familiarity only affected pupil dilation for the voice that was trained for the longest, and we found no evidence for reduced pupil dilation for the two voices that were trained for shorter lengths of time, when compared to unfamiliar voices. These results appear to suggest that the two putative measures of effort—self-report and physiological—may be differentially sensitive to the extent of voice familiarity, with listeners being able to notice that they are exerting less effort even for voices they have been trained on for approximately 10 min, whereas physiological measures do not show differences unless a voice has been trained for approximately 1 h. Although, it is important to note that the interaction between Target Familiarity and Measurement was not significant. Therefore, rather than reflecting a real difference in how voice familiarity is reflected in the two measures, the apparent differences could instead reflect greater noise for the effect of voice familiarity on pupil recordings compared to the effect of voice familiarity on self-report scores. Alternatively, there may be a real difference in how voice familiarity is reflected in the two measures, but it has a sufficiently small effect size that is difficult to detect with a sample size of 20 participants.

Despite finding no significant interaction between Familiarity and Measurement, we did find a significant interaction between TMR and Measurement, which indicates that the two effort measures are differentially sensitive to TMR. TMR had a significant effect on self-reported effort, but not on pupil diameter. We found a trend in the expected direction for pupil diameter (Zekveld et al., 2018), but the difference between the two TMRs was not significant. Thus, it is possible that self-reported effort is more sensitive than pupil dilation to differences in TMR. Although, another possible explanation is that effects on self-reported effort could be affected by response bias—for example, due to demand characteristics (Orne, 1962). In other words, participants may not have experienced less effort in these conditions, but reported less effort because they noticed differences between conditions and believed they should report effort differently across conditions. Or, participants could have been reporting difficulty or perceived performance, rather than effort (McGarrigle et al., 2014). In our study, the researcher explained to participants the differences between effort, difficulty, and performance at the beginning of the speech-intelligibility test, thereby reducing the likelihood that listeners’ ratings of effort were conflated with difficulty or performance. Nevertheless, the inherent nature of a self-report measure makes it difficult to fully rule out these possibilities.

A similar pattern of results, that self-reported effort is more sensitive than pupil diameter to TMR, was also reported by Wendt et al. (2016); although, the opposite pattern was found by McGarrigle et al. (2017). Therefore, the effects may be sensitive to the specific TMRs and types of competing sounds used (Johns et al., 2024; Zekveld et al., 2018). Notably, the difference between the two TMRs we used here—of +3 dB and −6 dB—is smaller than those used in some previous studies (e.g., McGarrigle et al., 2017), which could explain differences in the results. Regardless, it is not too surprising that these two measures gave different results, given that physiological and self-report measures of listening effort often diverge (McGarrigle et al., 2014). Possibly, these measures may index different underlying cognitive or neurobiological processes, consistent with multidimensional views of listening effort (Alhanbali et al., 2019; Shields et al., 2023). For example, they may index different components associated with listening effort, such as general arousal, engagement in the specific task, evaluating task demands, allocating resources, or the motivation to overcome demands (Pichora-Fuller et al., 2016; Saderi et al., 2021); or, they be differentially sensitive to the type of demand that underpins increased effort—for example, increased effort may arise from demands on attention, working memory, or speed of processing, which have overlapping but dissociable neurobiological substrates (Friedman et al., 2008; Gajardo-Vidal et al., 2024; Mendoza-Halliday et al., 2024). Further work comparing putative listening-effort measures under different scenarios is needed to elucidate the reasons for the differences observed in the current and previous studies.

Our finding that trained voices were more intelligible than unfamiliar voices (Figure 5) replicates previous work (Case et al., 2018; Holmes et al., 2021; Kreitewolf et al., 2017; Levi, 2015; Levi et al., 2011; Nygaard et al., 1994; Nygaard & Pisoni, 1998; Yonan & Sommers, 2000; Zhu & Holmes, 2025). Although, we found different effects of familiarity with the target voice at the two TMRs: We found a significant effect at +3 dB, but not at −6 dB. This finding contrasts with previous assumptions that familiarity has the greatest effect on speech intelligibility in less favorable acoustic conditions (e.g., Johnsrude et al., 2013; Yonan & Sommers, 2000; Zhu & Holmes, 2025). Yet, it is consistent with the results of another study (in preparation) using the same TMRs, in which we also found the biggest familiar-voice benefit at +3 dB TMR and a smaller (but nevertheless significant) effect of familiarity at −6 dB TMR. The reason why familiar-voice effects are strongest at different TMRs across studies is unclear; although, it is unlikely to be explained by poor performance in the task at the lower TMR, because participants were still performing well above chance (0.02%) for unfamiliar voices at −6 dB TMR (∼35%). Another possible explanation is that acoustic masking at the lower TMR obscures cues to familiarity, although this explanation is also unlikely given that previous studies have found familiar-voice benefits at −6 dB TMR (e.g., Domingo et al., 2020; Holmes et al., 2021; Johnsrude et al., 2013; Zhu & Holmes, 2025). Also, interestingly, in contrast to the effects on speech intelligibility, we found no evidence that magnitude of the reduction in self-reported effort or pupil dilation for familiar voices differed between the two TMRs. Ultimately, future studies that test a wider variety of TMRs are needed to examine this question further.

Nevertheless, the overall magnitude of the familiar-voice benefit that we observed here (13.8%, on average) is consistent with previous research: the present study replicates the voice training paradigm of Holmes et al. (2021), who found a speech-intelligibility benefit of 7%–15% (9.31%, on average). The magnitude is also comparable to that reported in studies which utilize the same task and type of masker to investigate the familiar talker advantage, but where listeners hear naturally familiar voices (Domingo et al., 2020; Johnsrude et al., 2014). This result reinforces the idea that familiar voices are more intelligible than unfamiliar voices, even following short durations of lab-based training. This effect is not simply due to familiarity with the sentence materials themselves, as we used different sentence materials during the training and test phases of the experiment—meaning that the benefit reflects familiarity with a voice that generalizes to new sentence materials.

Unlike Holmes et al. (2021), we found no significant differences in speech intelligibility between the three voices that were trained for different lengths of time. Holmes et al. (2021) found a similar-magnitude benefit for the two voices that were trained for the shortest lengths of time, and a significantly bigger benefit for the voice that was trained for the longest, but we did not replicate this effect in the current study. One possible reason may be that we had a smaller sample size (20 compared to 50 participants), which would be less sensitive to differences in the magnitude of the familiar-voice benefit among the three voices. Despite not finding a difference in speech intelligibility between the three voices, we did find a difference in pupil diameter, which provides evidence that the voice trained for the longest was perceived differently to the voices that were trained for shorter durations.

The current study is the first, to our knowledge, that has examined whether voice familiarity reduces effort when listeners try to understand speech in competing speech. A previous study by Biçer et al. (2023) studied how voice training effects pupil dilation during a voice-cue discrimination task, in which participants discriminated differences in fundamental frequency and vocal tract length among three-syllable consonant-vowel stimuli. Similar to our results, they found that trained voices elicited less pupil dilation than unfamiliar voices, although their effects were subtle. They did not find a significant effect on mean pupil dilation or other summary measures (e.g., peak dilation or latency) and they only found differences in a nonlinear Generalized Additive Mixed Models analysis. In that analysis, they also only found differences in pupil responses when stimuli were vocoded and no differences when the stimuli were not vocoded. Our results may help to explain this subtle effect, as we only found significant effects on mean pupil dilation for the voice that was explicitly trained for ∼1 h and not for voices that were trained for shorter lengths of time. Biçer et al. (2023) trained voices implicitly by asking participants to listen to an audiobook lasting 30 min. Possibly, if they had used longer durations of training, and explicit training with feedback, they may have observed stronger effects on pupil responses.

Some researchers have argued that apparent reductions in effort may be conflated with performance (e.g., Moore & Picou, 2018). In other words, when participants are asked about effort, they are really answering a question about how well they think they performed. Similarly, reduced pupil dilation could reflect better speech intelligibility when performance is not matched between conditions. However, there are now many reported cases where performance and effort measures do not match, and are dissociable (Winn & Teece, 2021). Here, we reduced the likelihood that participants were self-reporting performance by explaining the difference to participants before they began (see Supplemental Material). In addition, we found distinct patterns of results across performance, self-reported effort, and pupil diameter measures: For example, we found a significant effect of TMR on accuracy and self-reported effort, but not on pupil diameter, and we found a significant interaction between TMR and Familiarity for accuracy, but not for self-reported effort or pupil diameter. Together, these results suggest that the measures are not entirely conflated with one another.

Our finding that voice training improves intelligibility and reduces effort is promising for real-world interventions designed to improve speech understanding and reduce fatigue when listening in noisy environments. Noisy environments are challenging for many people, although may be particularly difficult for older adults and people with hearing loss (Duquesnoy, 1983; Marrone et al., 2008), for whom exerting a high level of listening effort throughout the day can lead to excessive fatigue (Alhanbali et al., 2017; Hornsby et al., 2016). If individuals could train themselves on the voices that they are likely to hear in noisy, everyday situations, it could make listening to speech more accurate and less effortful, allowing them to communicate successfully in noisy environments and reach the end of the day feeling less fatigued.

In conclusion, our results underscore the importance of voice familiarity in speech perception, showing advantages for both speech intelligibility and listening effort after relatively short (≤1 h) durations of lab-based training. In addition, our results demonstrate differing sensitivities of two widely used measures of listening effort—self-report scores and pupil dilation.

## Supplemental Material

sj-docx-1-tia-10.1177_23312165251401318 - Supplemental material for Voice Familiarization Training Improves Speech Intelligibility and Reduces Listening Effort Supplemental material, sj-docx-1-tia-10.1177_23312165251401318 for Voice Familiarization Training Improves Speech Intelligibility and Reduces Listening Effort by Freja Baxter, Harriet J. Smith and Emma Holmes in Trends in Hearing
