# Supplementary material for: Voice Familiarization Training Improves Speech Intelligibility and Reduces Listening Effort
Source: Trends Hear. 2025 Dec 8;29:23312165251401318. doi: 10.1177/23312165251401318 (PMC12686366; doi:10.1177/23312165251401318)
Supplement: sj-docx-1-tia-10.1177_23312165251401318 - Supplemental material for Voice Familiarization Training Improves Speech Intelligibility and Reduces Listening Effort [file sj-docx-1-tia-10.1177_23312165251401318.docx]

Listening effort

After each sentence, you will be asked to rate your effort on a scale from 1 to 7:

1 2 3 4 5 6 7


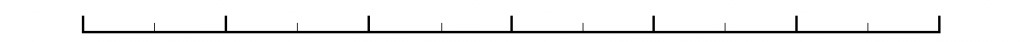


no effort very little little moderate considerable very much extreme

effort effort effort effort effort effort

Please try to use the full scale to show how your effort varies and try not to choose the same number every time

Important: Your effort rating should reflect *how hard you worked* to get to the answer you selected, and NOT how difficult it was or how well you think you did.

Sometimes, you might put in a lot of effort but still not get it correct. Other times, you might put in very little effort and not get the answer correct.

Other times, you might choose the correct answer but you didn’t need to put in much effort. Whereas, sometimes you might reach the correct answer but find you needed to exert a lot of effort to get there
